# Developmental origins of psycho-cardiometabolic multimorbidity in adolescence and their underlying pathways through methylation markers: a two-cohort study

**DOI:** 10.1007/s00787-024-02390-1

**Published:** 2024-02-17

**Authors:** Priyanka Choudhary, Justiina Ronkainen, Jennie Carson, Ville Karhunen, Ashleigh Lin, Phillip E. Melton, Marjo-Riitta Jarvelin, Jouko Miettunen, Rae-Chi Huang, Sylvain Sebert

**Affiliations:** 1 Research Unit of Population Health, Faculty of Medicine, University of Oulu, Oulu, Finland; 2 Telethon Kids Institute, Perth, Australia; 3 School of Population and Global Health, University of Western Australia, Perth, Australia; 4 Research Unit of Mathematical Sciences, Faculty of Science, University of Oulu, Oulu, Finland; 5 UWA Centre for Child Health Research, University of Western Australia, Perth, Australia; 6 Menzies Institute for Medical Research, University of Tasmania, Hobart, TAS Australia; 7 Department of Epidemiology and Biostatistics, School of Public Health, Imperial College London, London, UK; 8 MRC-PHE Centre for Environment and Health, School of Public Health, Imperial College, London, UK; 9 Department of Life Sciences, College of Health and Life Sciences, Brunel University London, Kingston Lane, Uxbridge, Middlesex UK; 10 Medical Research Center Oulu, Oulu University Hospital and University of Oulu, Oulu, Finland; 11 School of Medical and Health Sciences, Edith Cowan University, Perth, WA Australia; 12 Nutrition and Health Innovation Research Institute (NHIRI), School of Medical and Health Sciences, Edith Cowan University, Perth, WA Australia

**Keywords:** Adolescents, DNA methylation age, Epigenetics, Multimorbidity, Psycho-cardiometabolic

## Abstract

Understanding the biological mechanisms behind multimorbidity patterns in adolescence is important as they may act as intermediary risk factor for long-term health. We aimed to explore relationship between prenatal exposures and adolescent’s psycho-cardiometabolic intermediary traits mediated through epigenetic biomarkers, using structural equation modeling (SEM). We used data from mother–child dyads from pregnancy and adolescents at 16–17 years from two prospective cohorts: Northern Finland Birth Cohort 1986 (NFBC1986) and Raine Study from Australia. Factor analysis was applied to generate two different latent factor structures: (a) prenatal exposures and (b) adolescence psycho-cardiometabolic intermediary traits. Furthermore, three types of epigenetic biomarkers were included: (1) DNA methylation score for maternal smoking during pregnancy (DNAmMSS), (2) DNAm age estimate PhenoAge and (3) DNAm estimate for telomere length (DNAmTL). Similar factor structure was observed between both cohorts yielding three prenatal factors, namely BMI (Body Mass Index), SOP (Socio-Obstetric-Profile), and Lifestyle, and four adolescent factors: Anthropometric, Insulin-Triglycerides, Blood Pressure, and Mental health. In the SEM pathways, stronger direct effects of F1_prenatal_-BMI (NFBC1986 = *β*: 0.27; Raine = *β*: 0.39) and F2_prenatal_-SOP (*β*: −0.11) factors were observed on adolescent psycho-cardiometabolic multimorbidity. We observed an indirect effect of prenatal latent factors through epigenetic markers on a psycho-cardiometabolic multimorbidity factor in Raine study (*P* < 0.05). The present study exemplifies an evidence-based approach in two different birth cohorts to demonstrate similar composite structure of prenatal exposures and psycho-cardiometabolic traits (despite cultural, social, and genetic differences) and a common plausible pathway between them through underlying epigenetic markers.

**Supplementary Information:**

The online version contains supplementary material available at 10.1007/s00787-024-02390-1.

## Introduction

People with poor physical health often experience concurrent mental health concerns [1]. Much of the attention in this area is focused on adults, when cardiometabolic health outcomes become more apparent. However, upcoming evidence suggests that cardiometabolic risk can be identified at younger age, thus affording the opportunity to intervene to prevent comorbidities [2]. While gestation is an important developmental phase, adolescence is a critical transition period, characterized by rapid and ordinated developmental stages, only second to early childhood in its rate and breadth of effect upon mental and biological health concerns [3, 4].

The risk is posited to spiral from one generation to another, arising from adverse biological and environmental factors during gestation. Various in utero adversities together have immediate consequences on birth outcomes and are also known to influence the future burden of psycho-cardio-metabolic disorders [5–7]. Along these lines, in our previous study, we identified inverse association between maternal prenatal biopsychosocial score and birth weight [8], which is a known risk factor for numerous diseases across the lifespan [9]. In the study, four biopsychosocial latent factors, namely Factor1-BMI, Factor2-DBP (Diastolic Blood Pressure), Factor3-Socioeconomic-Obstetric-Profile (SOP), and Factor4-Parental-Lifestyle were derived using multiple in utero measures and the factor scores from each latent factor were combined into a cumulative score.

The link between heritability and environmental determinants of psycho-cardiometabolic traits, such as glucose intolerance, lipids, anxiety, and depression, is commonly explained through the epigenetic variability related to influences of the early life adversities as one of the plausible mechanisms [10, 11]. One such environmental determinant is maternal smoking during pregnancy, a well-established early life exposure strongly associated with the epigenetic variability in offspring until later life [12–14]. These differential epigenetic changes also mediate the association of prenatal maternal smoking on lower birth weight [15] and were subsequently shown to have long-term association with cardiometabolic intermediary traits [13] and psycho-cardiometabolic disorders [16]. Moving forward our research group has generated and evaluated a novel DNA methylation (DNAm) risk score in adolescents aiming to predict fetal exposures to maternal smoking during pregnancy (DNAmMSS: DNAm maternal smoking score during pregnancy) [17] and evaluate their subsequent risk upon future cardiometabolic diseases. This offers insight on the underlying paradigm of ‘biological embedding’ to diseases and may enable identifying individuals at risk of unknown exposures.

Epigenetic signatures using DNAm levels at age-related DNAm sites have been developed to capture aspects of biological aging. These DNAm age estimates vary within individuals of the same chronological age based on the incidence of age-related chronic diseases [18]. Several DNAm age estimates have been developed and among them PhenoAge is a score using 513 CpG sites to estimate “phenotypic age” derived from a set of clinical biomarkers of aging and it correlates well with chronological age [19]. Telomere Length (TL) is another important marker of biological age, as average TL decreases with age. Lu et al. developed a DNA methylation estimator of TL (DNAmTL: DNAm estimate for TL) based on 140 CpGs which was related to age-related pathologies [20]. Both PhenoAge and DNAmTL are observed to perform better than other DNAm scores in detecting the association with age, sex, behavioral factors, and numerous clinical intermediary traits as well as intrauterine exposures [19, 20].

Based on these previous studies [8, 17], this study aims to further expand on modeling latent constructs of prenatal exposures and adolescent’s psycho-cardiometabolic intermediary traits to test the pathways between them using different intermediary epigenetic signatures. Our specific objectives are to: (1) develop prenatal exposure and adolescent psycho-cardiometabolic comorbidities latent construct; (2) identify shared pathways from early life leading to adolescent psycho-cardiometabolic multimorbidity using epigenetic biomarkers: DNAmMSS, PhenoAge, and DNAm TL; and (3) cross-validate the findings between two birth cohorts: Northern Finland Birth Cohort 1986 (NFBC1986) and the Raine Study.

## Methods

The data were derived from two prospective cohorts: NFBC1986 and the Raine Study. The NFBC1986 is a pregnancy-birth cohort consisting of 99% (*N* = 9215) of all children born in the recruitment zone (provinces of Oulu and Lapland) in the Northern Finland between 1 July 1985 and 30 June 1986 [21]. Offspring have been followed up until the age of 16 years, and the data were collected through a clinical examination (*n* = 5654) and postal questionnaires (*n* = 7344).

The Raine Study is a longitudinal Western Australia Pregnancy Cohort established in 1989 [22, 23]. From 1989 to 1991, pregnant women (*N* = 2900, Gen1) were recruited at King Edward Memorial Hospital and surrounding private hospitals. Of which 2,868 live births (Gen2) are followed up from 18 weeks’ gestation into young adulthood at multiple time points in order to investigate the early origins of adult disease through anthropometric, clinical, biochemical, and questionnaire data.

The present study included participants from singleton births and with complete data at each time point. Availability of DNA methylation data was the main reason for attrition in the sample size.

### Measures

#### Prenatal exposures

In the NFBC1986, antenatal data were collected at 12th, 20th, and 36th week of pregnancy and in the Raine Study from antenatal visits at 18th and 34th week of gestation. In both cohorts, maternal pre-and-end pregnancy body mass index (BMI-kg/m^2^) was calculated through height and weight measured at the time of enrollment and at 36th/34th week of gestation. Maternal age, marital status, parity, alcohol use, and maternal and paternal smoking were self-reported through questionnaire. The measures were coded into dichotomous variables as: ‘*married* and *unmarried* (including single and widow)’; ‘*nulliparous* and *multiparous*’; ‘*any maternal smoking during pregnancy* and *no smoking during pregnancy’*; father smoking: ‘*yes* or *no*’; ‘*any maternal alcohol use during pregnancy* and *no alcohol use’*.

#### Epigenetic mediators

In the NFBC1986, DNA was extracted from all 5,654 whole blood samples available at the 16-year follow-up. Of these, DNA methylation for 546 randomly selected participants with complete follow-up data available was measured on Illumina Infinium HumanMethlation450K array (Illumina, San Diego, USA) at the Department of Genomics Imperial College London (London, UK). After quality control and based on variable availability, 490 samples were used in the analyses [16]. In Raine Study, at the 17-year follow-up, DNA methylation was measured in peripheral whole blood sample of 996 European ancestry participants using the same Illumina Infinium HumanMethylation450K BeadChip [24]. We included three epigenetic scores in this study: DNAmMSS [17], DNAm age estimate PhenoAge [19], and DNAmTL [20]. DNAmMSS was developed as a proxy measure for exposure to maternal smoking during pregnancy by Rauschert et al. [17]. The score was developed with 204 CpGs using elastic net regression. It was first tested in the Raine Study using tenfold cross-validation and then validated independently in NFBC1986. PhenoAge was developed using 513 CpG sites from whole blood of adults [19]. It was trained on a chronological age-based composite clinical phenotypic measures of age, including nine biomarkers: albumin, creatinine, glucose, lymphocytes, C-Reactive Protein, mean cell volume, red cell distribution, alkaline phosphate, and white blood cell counts. The used intermediary measure was calculated as the residuals from the regression of DNAm age and chronological age. Among different measures of DNAm age scores, such as Hannum [25], Horvath [26], Horvath’s estimate for skin and blood [27], we included PhenoAge in our study as it was strongly correlated with chronological aging. PhenoAge is generated using different metabolic and aging markers, thus more closely representing biological health variance. DNAmTL is based on 140 CpGs and applicable over the entire age spectrum. It is considered more robust than Leucocyte TL and outperformed in detecting the association of age, sex, behavioral factors, with numerous clinical intermediary traits [20].

#### Adolescent outcomes

The adolescent measures were comparable in both cohorts, available at 16 years in NFBC1986 and at 17 years in the Raine Study. In both the studies, clinical examination was carried out to estimate the anthropometric and cardio-metabolic traits. Height, weight, and WC were measured by a nurse. Height and weight were converted to BMI as kg/m^2^ in the current study. BP was measured twice with ten minutes apart and the average of the measurements was used. Blood samples were extracted after overnight fasting to measure fasting glucose (mmol/l), insulin (mmol/l) and lipids (mmol/l) measures. The information on psychological symptoms was collected using Youth Self-Report scale [28]. In this study we included three sub-scales including questions that indicate anxious-depressed, withdrawn depressed and somatic complaints (supplementary methods, additional file 1).

### Statistical analysis

#### Factor analysis

We employed a latent variable approach which is robust to measurement error and allows for variable reduction. Two different latent factor structures were generated: one for prenatal exposures and another for adolescent psycho-cardiometabolic intermediary traits. The prenatal latent factors were derived based on a previous study conducted in NFBC1986 [8]. Adolescent psycho-cardiometabolic latent factors were modeled independently in each cohort. We used Mplus 7.0 employing EFA to first identify the structure for adolescent psycho-cardiometabolic traits and confirmatory factor analysis (CFA) to confirm the final model for both prenatal and adolescent factors [29]. The analysis used weighted least squares mean and variance adjusted parameter estimates which is appropriate for categorical variables and geomin oblique rotations for correlations between the factors. The factorial structure was determined using model fit indices: RMSEA < 0.06, CFI > 0.90, and TLI > 0.90 [30, 31]. Factor scores were extracted (continuous values with mean = 0 and SD = 1) for each latent factor to use in the subsequent analysis.

#### Correlation matrix

We used Pearson correlation to identify the correlation matrix between the following variables: prenatal latent factors, adolescent psycho-cardiometabolic latent factors, DNAmMSS, PhenoAge and DNAmTL.

#### Structural equation modeling

Using AMOS v7 [32], all the measures were combined in a structural equation model (SEM) to investigate the proposed pathways in both cohorts separately. A common multimorbidity latent factor from the four adolescent psycho-cardiometabolic co-morbidities factors was created using second-order factor approach. This allowed us to look at the relationship of each of the components and to test our hypothesis that an interplay of adversities from early life are modulated through epigenetic markers leading to shared pathways to multimorbidity in adolescence. The prenatal latent factors included in this study have been tested previously for their relationship with birth weight. Hence, birth weight was not included in the model.

## Results

The complete case sample sizes were 490 for NFBC1986 and 995 for the Raine Study (Table 1). In comparison to NFBC1986, the Raine Study mothers were more often unmarried/single/separated, nulliparous and their smoking and alcohol use were higher during pregnancy. Among adolescent measures, NFBC1986 adolescents had higher fasting glucose, insulin, and diastolic blood pressure and the Raine Study had higher BMI (Body Mass Index), WC (Waist Circumference), and triglycerides levels.

**Table 1:** Study population characteristics in NFBC1986 and Raine Study

	NFBC1986 (Methylation sample *n* = 490)		Raine study (Methylation sample *n* = 995)	
	Observations (*N*)	Mean (SD)/Median (IR) or *n* (%)	Observations (*N*)	Mean (SD)/Median (IR) or *n* (%)
*Parental measures during pregnancy*				
Maternal pre-pregnancy BMI (kg/m^2^)	490	21.7(±3.5)	870	22.5 (±4.5)
Maternal end-pregnancy BMI (kg/m^2^)	490	27.1 (±4)	946	27.6 (±4.6)
Maternal age (years)	490	28 (±7)	971	28.9 (±5.8)
Marital status (Unmarried/single)	465	15 (2.8%)	995	120 (12.1%)
Nulliparous	461	173 (32.8%)	764	367 (48%)
Maternal smoking	483	31 (6.4%)	995	232 (23.4%)
Maternal alcohol use	487	54 (10.3%)	995	489 (49.2%)
Paternal smoking	490	169 (34.5%)	990	354 (35.7%)
*Offspring measures at adolescence*				
Sex (Females)	490	293 (54.1%)	995	495 (49.6%)
BMI (kg/m^2^)	490	20.6 (±3.7)	747	22.2 (±4.6)
Waist Circumference (cm)	489	72 (±10.1)	716	77 (±12.2)
Insulin (mmol/l)	487	10 (±5.5)	852	7.7 (±5.7)
Triglycerides (mmol/l)	490	0.7 (±0.5)	852	0.9 (±0.5)
HDL-C (mmol/l)	487	1.4 (±0.4)	875	1.3 (±0.4)
Systolic BP (mmHG)	490	115 (±12)	744	114 (±10)
Diastolic BP (mmHG)	490	68 (±9.5)	744	59 (±6)
Anxious-depressed	484	15 (±4)	715	16 (±5)
Withdrawn depressed	464	9 (±3)	719	10 (±4)
Somatic complaints	460	13 (±4)	708	13 (±5)
Smoking	490	174 (35.5%)	720	162 (22.5%)

*BMI* body mass index, *HDL-C*: high density lipid—cholesterol

Note: Values are percentages for categorical variables, mean (±SD) for continuous variables with normal distribution and median (IR) for skewed variables

### Latent factors

**Fig. 1:**
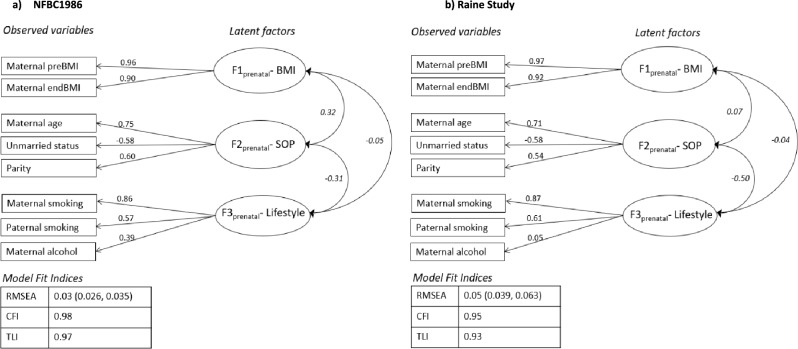
Confirmatory factor analysis model of prenatal exposures in **a** NFBC1986 and **b** Raine Study. *Boxes* represent observed variables; *circles* represent latent factors and *two-way arrows* represent correlation between factors. Pearson correlation coefficients are written in *italics*. *Values* represent factor loadings of observed variables on latent factor. *BMI* body mass index, *SOP* socio-obstetric-profile

A similar three-factor structure supported by CFA with all measures loading strongly onto their respective prenatal latent factors in both cohorts (NFBC1986 = RMSEA (Root Mean Square Error of Approximation): 0.03, CFI (Comparative Fit Index): 0.98, TLI (Tucker Lewis Index): 0.97; Raine Study = RMSEA: 0.05, CFI: 0.98, TLI: 0.97) (Fig. 1). The first factor characterized by pre-and-end pregnancy BMI was labeled as ‘F1_prenatal_-BMI’. The second factor was labeled as ‘F2_prenatal_-Socioeconomic-Obstetric-Profile (SOP)’ representing ‘parity’, ‘maternal age’ and ‘unmarried status’. The third factor characterized maternal and paternal smoking and maternal alcohol use, termed as ‘F3_prenatal_-Lifestyle’. Similar correlations between factors were observed in both cohorts, with strongest correlation between ‘F2_prenatal_-SOP’ and ‘F3_prenatal_-lifestyle’.

**Fig. 2:**
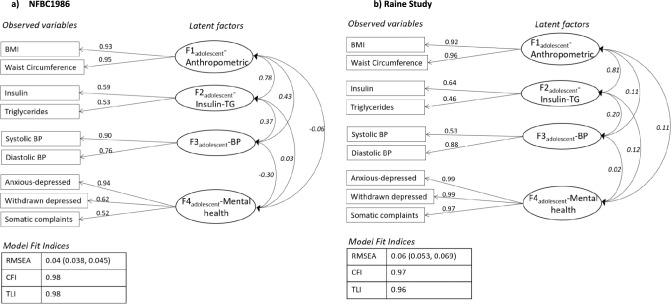
Confirmatory factor analysis model of adolescent comorbidities in **a** NFBC1986 and **b** Raine Study. *Boxes* represent observed variables; *circles* represent latent factors and *two-way arrows* represent correlation between factors. Pearson correlation coefficients are written in italics. *Values* represent factor loadings of observed variables on latent factor. *BP* blood pressure, *TG* triglycerides

For adolescent psycho-cardiometabolic traits, EFA (Exploratory Factor Analysis) yielded four-factor structure displaying distinct biological and psychological groupings (Supplementary Table S1, Additional File 1). This was further supported by the CFA (Fig. 2) and the fit statistics, structures and factor loadings were comparable across both cohorts (NFBC1986 = RMSEA: 0.04, CFI: 0.98, TLI: 0.98; Raine Study = RMSEA: 0.06, CFI: 0.97, TLI: 0.96). The factors were labeled to closely represent the included observed variables, for instance: ‘F1_adolescent_-Anthropometrics’ to characterize BMI and WC, ‘F2_adolescent_-InsulinTG’ to characterize insulin and triglyceride, ‘F3_adolescent_-BP’ to characterize systolic and diastolic BP, and ‘F4_adolescent_-Mental health’ to characterize anxious-depressed, withdrawn depressed and somatic complaints. Across both cohorts, the strongest correlations among the latent factors were observed between ‘F1_adolescent_-Anthropometrics’ and ‘F2_adolescent_-InsulinTG’ (Fig. 2). Differences were observed in the correlations of ‘F4-Mental health’ with other factors between the two cohorts.

### Correlations between latent factors and epigenetic biomarkers

Distinct correlation clusters and similarities were observed between variables across both cohorts (Supplementary Fig S1, Additional File 1). Most of the variables were significantly correlated (*P* < 0.001). Among maternal factors, ‘F1_prenatal_-BMI’ was most strongly correlated with adolescent factors. For epigenetic markers, DNAmMSS showed strongest correlated with ‘F2_prenatal_-SOP’ and ‘F3_prenatal_-Lifestyle’, followed by DNAmTL which was inversely correlated with most of the factors with exception of ‘F1_prenatal_-BMI’, ‘F2_prenatal_-SOP’ and ‘F4_adolescent_-Mental health’.

### SEM

**Fig. 3:**
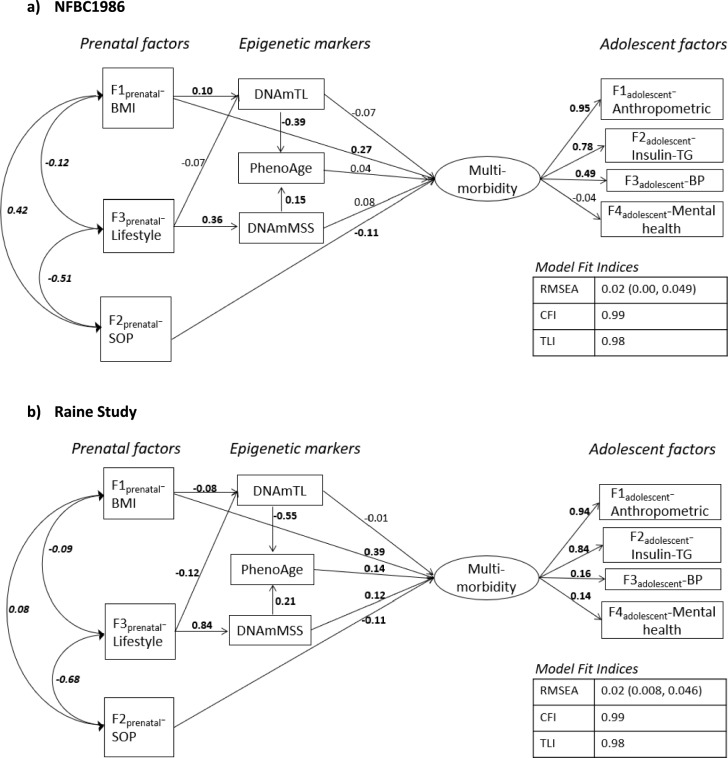
Structural equation modeling pathways in **a** NFBC1986 and **b** Raine Study. The values are standardized regression coefficients of direct effect. Values with *P* < 0.05 are denoted with bold fonts and *P* > 0.05 with normal font. *BP* blood pressure, *BMI* body mass index, *DNAmMSS* DNA methylation maternal Smoking Score, *SOP* socio-obstetric-profile, *TG* triglycerides, *TL* telomere length

An overview of the path model in accordance with our hypothesis is shown in Fig. 3, displaying comparable pathways in both cohorts. The fit indices for the multilevel SEM indicated a good model fit for both the cohorts (NFBC1986 = RMSEA: 0.03, CFI: 0.99, TLI: 0.98; Raine Study = RMSEA: 0.02, CFI: 0.99, TLI: 0.98). The psycho-cardiometabolic multimorbidity latent factor showed stronger representation of adolescent biological indicators than the mental health indicators. In the SEM pathways, stronger direct effects of the ‘F1_prenatal_-BMI’ (NFBC1986 = *β*: 0.27; Raine Study = *β*: 0.39) and ‘F2_prenatal_-SOP’ (*β*: −0.11) were observed on adolescent psycho-cardiometabolic multimorbidity factor. F3_prenatal_-Lifestyle showed only indirect effect (NFBC1986 = *β*: 0.04; Raine Study = *β*: 0.12 -Supplementary Table S2, Additional File 1) on the psycho-cardiometabolic multimorbidity factor. F3_prenatal_-Lifestyle had the strongest direct effect on the DNAmMSS (NFBC1986 = *β*: 0.36; Raine Study = *β*: 0.84). The indirect effect of the prenatal factors on multimorbidity through epigenetic markers was mediated from DNAmTL and DNAmMSS going through PhenoAge in Raine study (*P* < 0.05).

## Discussion

The current study is novel in providing an overview of the shared molecular pathways arising from in utero adversities on different psycho-cardiometabolic measures as a multimorbidity in adolescents. Importantly, we did a cross-cohort comparison of our findings from distinctly independent birth cohorts from two culturally diverse countries (Finland and Australia). We observed both direct and indirect effects of prenatal latent factors on adolescent psycho-cardiometabolic multimorbidity through composite epigenetic scores, displaying the importance of epigenomes on later health outcomes. Our objective was to further expand on the understanding of the structure of prenatal adversities and mental and physical health especially in adolescence which has been lacking from the existing literature.

We observed distinct characteristics between the two cohorts. Although, both cohorts were recruited at the same time point, maternal smoking and alcohol use was much higher in Raine compared to NFBC1986. Moreover, cardio-metabolic measurements varied between the two cohorts, indicating health differences between the two cohorts. In line with our previous findings [8], the prenatal latent factor model fitted the data well and revealed similar correlations between both cohorts. A distinct pattern was observed for adolescents’ psycho-cardiometabolic traits, showing separate biological and psychological groupings (Fig. 2), as seen in a previous study [33]. Importantly, similar structure and factor loadings were observed for both cohorts when constructed independently. While, biological patterns were quite comparable among each other, heterogeneity was observed between ‘F4_adolescent_-Mental health’ and its correlation with other factors across the cohorts. For NFBC1986, ‘F4_adolescent_-Mental health’ was largely represented by anxious-depressed measure, and it was negatively correlated with ‘F1_adolescent_-Anthropometric’ and ‘F3_adolescent_-BP’. On the other hand, in the Raine Study, ‘F4_adolescent_-Mental health’ was equally characterized by each psychological symptom subscale (anxious-depressed, withdrawal depressed and somatic complaints) and was positively correlated with other latent factors. This suggests that the biological parameters behave similarly between different populations but not psychological aspects. The reason may be that these patterns in adolescent period behave differently from adult patterns; attributable to their rapid hormonal changes, and wide range of biological, psychological and social challenges occurring in adolescence phase [34]. Additionally, these are culturally, socially and genetically different populations from different continents, where dynamics of perceiving health may also vary largely [35].

Our multimorbidity second-order factor sheds important insights on the relationship between and with psycho-cardiometabolic traits in its entirety. The factor structure was largely representative of biological measures and less of mental states. This was expected as the correlations between biological factors and mental states were weak in the CFA model. Despite the imbalance, it is very interesting to note that all the psycho-cardiometabolic comorbidity factors loaded into one factor that replicates between cohorts. Individuals with mental health problems have up to 14 years of shorter life expectancy, which is often partly accounted by the co-occurring physical diseases [36]. Moreover, heritability studies suggests that the causes of multimorbidity have both genetic and environmental components shared between physical and mental disorders [37, 38]. Therefore, it was worthwhile to unravel the shared relationships between psycho-cardiometabolic multimorbidity, which is not captured when looking at the traits individually.

Together, our SEMs revealed plausible pathways to multimorbidity. Specifically, among prenatal latent factors, ‘F1_prenatal_-BMI’ had the strongest direct influence on adolescent psycho-cardiometabolic multimorbidity. Maternal BMI embodies both a biological dimension as well as lifestyle and social factors, such as maternal age, marital status, smoking, and alcohol use [39]. These correlations were also reflected in our correlation matrix (Supplementary Fig S1, Additional File 1).

In the same way, ‘F2_prenatal_-SOP’ showed a direct effect on multimorbidity, but here the direction was negative, and no effect was modulated through epigenetic factors. This suggests that not all early life influences have epigenetic influence, particularly social factors (maternal age, marital status, parity). Additionally, the negative effect on the psycho-cardiometabolic multimorbidity factor highlights the protective dimension of social factors, such as decreased parity, younger maternal age, and married status. The ‘F3_prenatal_-Lifestyle’, while not showing a direct effect, showed a strong indirect effect in Raine study, primarily through DNAmMSS. Its strong intermediary role from ‘F3_maternal_-Lifestyle’ in our pathway analysis (Fig. 3) confirms the validity of the score as a proxy of ‘in utero* adversity*’ since it mirrored the known association of prenatal smoking-related epigenetic changes on cardio-metabolic health of the offspring in previous observational studies [13, 14, 16].

Epigenetic markers are important molecular readout of diverse environmental exposures across the lifespan. In our study, we observed that DNAmTL and DNAmMSS showed direct as well indirect influence going through PhenoAge marker. Increasing evidence supports the concept of molecular aging as a component of chronic diseases and an important tool for predicting biological age of an individual [18]. Biological age evaluated using these epigenetic markers has been shown to vary within individuals of same chronological age based on the incidence of chronic mental and physical diseases [40] and is also significantly influenced by intrauterine conditions [41]. DNAmTL showed negative relationship with all the path variables in our study. Telomere length is largely determined already during early fetal development and associates with several maternal factors during pregnancy, including maternal smoking, stress, socioeconomic status, BMI and gestational diabetes [42]. It is speculated that shorter TL may weaken the replicative potential and diminish somatic repair contributing to degenerative diseases such as cardio-metabolic diseases [43]. Correspondingly, our findings regarding PhenoAge were consistent with previous studies in showing the association with cardiometabolic risk factors. Importantly, it was observed to mediate the indirect effect of all the other epigenetic biomarker path factors particularly in Raine Study, highlighting its importance on phenotypic outcomes. Studies from both European and African American cohorts have reported association of PhenoAge with a wide range of phenotypes, such as smoking, blood pressure, insulin, glucose, triglycerides, and low-density lipid cholesterol [19, 44]. Nonetheless, PhenoAge is relatively new DNAm age estimate, so further replication is required to fully understand its association with a range of health outcomes. However, we observed in our study that more than 20% adolescents with personal smoking status, hence it could be a potential confounder in the relationship between epigenetic markers and psycho-cardiometabolic traits [45, 46].

A further point to note is that in both prenatal biopsychosocial and in adolescent psycho-cardiometabolic constructs, metabolic factor (F1_maternal_-BMI and F1_adolescent_-Anthropometric) showed consistently strongest correlations and largest representation in the latent factors. Thus, suggests that in our study adiposity, a potentially modifiable factor, had a predominant role over other predictors in defining adolescents’ health.

### Strengths and limitations

This is the first study to use a factor structure approach to examine the latent relationship between prenatal adversities factors and later adolescent psycho-cardiometabolic health. The benefit of using a factor structure instead of an individual measure of biological health or psychological status is that it allows us to account for the different aspects of these variables, represented by the sub-factors of commonality [30]. A further advantage is that the magnitude of factor loadings is determined empirically and does not comply with the assumption that all component measures have equal weighting.

We acknowledge the limitations of this study. In NFBC1986 methylation, sample size was much smaller than the full cohort sample. However, the characteristics of both samples were relatively comparable (Supplementary Table S3, Additional File 1). We included the most closely related and easily accessible prenatal measures as it can be challenging to develop a comprehensive model with maximum available measures, for which, we had limited similar prenatal measures harmonized between both cohorts. Mental health measures used in the study are subjective in nature and self-reported and might be under-reported leading to potential biases. There are other DNAm age estimates, such as Horvath [26], Hannum [25], and Horvath’s estimate for skin and blood [27], developed to predict biological age. However, in our study, DNAmTL and PhenoAge were more closely related to phenotypic markers than other DNAm age estimates. Both these markers were also recently developed and suggested to correlate better with mortality and morbidity [19]. A common limitation of biological age estimates is that they rely on specific organs or tissue; however, PhenoAge has been observed to relate with the wider range of tissue and cell types than other markers [19]. DNA methylation is a dynamic process influenced by multiple social, environmental, and lifestyle factors throughout the life course. In our study, both DNA methylation and psycho-cardiometabolic traits are measured at the same time point i.e., 16–17 years. Therefore, we cannot assume temporality, and it is plausible that there is reverse causation or bidirectional association between them. The mediated path coefficients observed in our study are small. Our study includes adolescent populations which are generally healthy and therefore the findings from this study should be interpreted in the same context and cannot be generalized to later ages.

## Conclusions

The present study exemplifies in two different cohorts similar composite structure of in utero maternal measures and psycho-cardiometabolic traits in adolescence, providing clarity on measures with cumulative risk. Our findings from cross-cohort analysis elucidate the differences in health between them and enhance the understanding of plausible common shared pathways from early life to psycho-cardiometabolic health through underlying epigenetic markers.

## Supplementary Information

Below is the link to the electronic supplementary material.

Supplementary file1 (DOCX 267 KB)

## Data Availability

NFBC data are available from the University of Oulu, Infrastructure for Population Studies. Permission to use the data can be applied for research purposes via electronic material request portal. In the use of data, we follow the EU general data protection regulation (679/2016) and Finnish Data Protection Act. The use of personal data is based on cohort participant’s written informed consent at his/her latest follow-up study, which may cause limitations to its use. Please, contact NFBC project center (NFBCprojectcenter@oulu.fi) and visit the cohort website (www.oulu.fi/nfbc) for more information.
