# Supplementary material for: Developmental origins of psycho-cardiometabolic multimorbidity in adolescence and their underlying pathways through methylation markers: a two-cohort study
Source: Eur Child Adolesc Psychiatry. 2024 Feb 17;33(9):3157–67. doi: 10.1007/s00787-024-02390-1 (PMC11424745; doi:10.1007/s00787-024-02390-1)
Supplement: Supplementary file 1 — Supplementary file1 (DOCX 267 KB) [file 787_2024_2390_MOESM1_ESM.docx]

**Additional File 1**

Table of Contents

[**Supplementary methods:** Youth Self-Report and their sub-scales used in the study 2](#_Toc145685449)

[**Table S1.** Exploratory factor analysis of adolescent psycho-cardiometabolic intermediary traits in NFBC1986 and Raine Study 3](#_Toc145685450)

[**Table S2.** The indirect and total effect values of SEM model for NFBC1986 and Raine Study 4](#_Toc145685451)

[**Table S3.** The study population characteristic of whole sample data and complete case data in NFBC1986 and Raine Study. 5](#_Toc145685452)

[**Fig S1.** Correlation between prenatal and adolescent psycho-cardiometabolic latent factors with epigenetic biomarkers: DNAmMSS, PhenoAge and DNAmTL. 6](#_Toc145685453)

# **Supplementary methods:** Youth Self-Report questions and their sub-scales used in the study.

The responses were scored as: 1 = Not true, 2 = Somewhat or sometimes true and 3 = Very true or often true.

**Sub-scale 1. Anxious/depressed**

I cry a lot

I am afraid of certain animals, situations or places, other than school

I am afraid of going to school

I am afraid I might think or do something bad

I feel that I have to be perfect

I feel that no one loves me

I feel worthless or inferior

I am nervous or tense

I am too fearful or anxious

I feel too guilty

I am self-conscious or easily embarrased

I think about seriously harming myself

I worry a lot

**Sub-scale 2. Withdrawn/depressed**

I would rather be alone than with others

I refuse to talk

I am secretive or keep things to myself

I am shy

I don´t have much energy

I am unhappy, sad or depressed

I keep from getting involved with others

**Sub-scale 3. Somatic complaints**

I have nightmares

I feel dizzy

I feel overtired

Physical problems without known medical cause: Aches or pains (not headaches)

Physical problems without known medical cause: Headaches

Physical problems without known medical cause: Nausea, feel sick

Physical problems without known medical cause: Problems with eyes

Physical problems without known medical cause: Rashea or other skin problems

Physical problems without known medical cause: Stomachaches or cramps

Physical problems without known medical cause: Vomiting, throwing up

# **Table S1.** Exploratory factor analysis of adolescent psycho-cardiometabolic intermediary traits in NFBC1986 and Raine Study

|  | **NFBC1986** | | | | **Raine Study** | | | |
| --- | --- | --- | --- | --- | --- | --- | --- | --- |
| ***Observed Variables*** | Factor 1 | Factor 2 | Factor 3 | Factor 4 | Factor 1 | Factor 2 | Factor 3 | Factor 4 |
| BMI | **0.91** | 0.25 | 0.00 | 0.01 | **0.95** | -0.02 | -0.00 | -0.01 |
| WC | **0.99** | 0.11 | 0.02 | -0.09 | **0.92** | 0.03 | 0.03 | 0.02 |
| Insulin | 0.29 | **0.95** | -0.03 | 0.07 | 0.23 | **0.39** | -0.04 | -0.02 |
| Triglycerides | 0.28 | **0.91** | 0.03 | 0.08 | 0.00 | **0.59** | 0.02 | 0.02 |
| Systolic BP | 0.02 | 0.03 | **1.35** | -0.09 | 0.00 | -0.00 | **1.17** | -0.00 |
| Diastolic BP | -0.00 | -0.00 | **0.47** | 0.09 | -0.12 | 0.14 | **0.42** | 0.00 |
| Anxious-depressed | -0.00 | -0.00 | -0.00 | **0.91** | -0.00 | 0.00 | -0.00 | **0.95** |
| Withdrawn depressed | -0.00 | -0.00 | 0.05 | **0.67** | 0.00 | 0.00 | 0.00 | **0.99** |
| Somatic complaints | 0.01 | 0.01 | 0.06 | **0.60** | 0.00 | -0.00 | 0.00 | **0.97** |
| **Model Fit Indices** |  | | | | | | | |
| Chi-sq | 821.32, df=30, *P*<0.001 | | | | 243.04, df=33, *P*<0.001 | | | |
| RMSEA | 0.056 (0.053, 0.059) | | | | 0.00 (0.00, 0.03) | | | |
| CFI | 0.95 | | | | 0.99 | | | |
| TLI | 0.96 | | | | 0.98 | | | |
| Note: Values are factor loadings for exploratory factor analysis using geomin rotations (*P*<0.001). Values in bold fonts represent factor loading greater than 0.3. Abbreviations: BMI: Body Mass Index; CI: Confidence Interval; BP: Blood Pressure; NFBC1986: Northern Finland Birth Cohort 1986, WC: Waist Circumference. | | | | | | | | |

# **Table S2.** The indirect and total effect values of SEM model for NFBC1986 and Raine Study

| **NFBC1986** | **Direct effect** | | **Indirect effect** | | **Total effect** | |
| --- | --- | --- | --- | --- | --- | --- |
| Path variables | PhenoAge | Multimorbidity | PhenoAge | Multimorbidity | PhenoAge | Multimorbidity |
| F1_prenatal_-BMI | 0 | 0.27 | -0.05 | -0.02 | -0.05 | 0.25 |
| F3_prenatal_-Lifestyle | 0 | 0 | 0.09 | 0.04 | 0.09 | 0.04 |
| DNAmMSS | 0.15 | 0.08 | 0 | 0.006 | 0.15 | 0.08 |
| DNAmTL | -0.39 | -0.07 | 0 | -0.02 | -0.39 | -0.09 |

| **Raine Study** | **Direct effect** | | **Indirect effect** | | **Total effect** | |
| --- | --- | --- | --- | --- | --- | --- |
| Path variables | PhenoAge | Multimorbidity | PhenoAge | Multimorbidity | PhenoAge | Multimorbidity |
| F1_prenatal_-BMI | 0 | 0.39 | 0.04 | 0.005 | 0.04 | 0.40 |
| F3_prenatal_-Lifestyle | 0 | 0 | 0.15 | 0.12 | 0.15 | 0.12 |
| DNAmMSS | 0.21 | 0.12 | 0 | 0.15 | 0.21 | 0.14 |
| DNAmTL | -0.55 | -0.01 | 0 | -0.07 | -0.55 | -0.06 |

Note: The values are standardized regression coefficients of indirect and total effect. Abbreviations: BMI: Body Mass Index; DNAmMSS: DNA methylation age maternal smoking score; DNAmTL: DNA methylation age telomere length; NFBC1986: Northern Finland Birth Cohort 1986.

# **Table S3.** The study population characteristic of whole sample data and complete case data in NFBC1986 and Raine Study.

|  | **NFBC1986** | | **Raine Study** | |
| --- | --- | --- | --- | --- |
|  | **Full sample**  ***n*=6764** | **Complete case sample**  ***n*=490** | **Full sample**  ***n*=1261** | **Complete case sample**  ***n*=990** |
| ***Offspring measures at adolescence*** | Mean (SD)/ Median (IR) or *n* (%) | Mean (SD)/ Median (IR) or *n* (%) | Mean (SD)/ Median (IR) or *n* (%) | Mean (SD)/ Median (IR) or *n* (%) |
| Sex (Females) | 3424 (50.6%) | 293 (54.1%) | 625 (49.6%) | 495 (49.6%) |
| BMI | 20.1 (+3.5) | 20.6 (+3.7) | 22.9 (+4.4) | 22.2 (+4.6) |
| Waist Circumference | 72 (+9) | 72 (+10.1) | 76 (+11.3) | 77 (+12.2) |
| Glucose | 5.2 (+0.5) | 5.2 (+0.5) | 4.7 (+0.6) | 4.7 (+0.5) |
| Insulin | 9.5 (+4.9) | 10 (+5.5) | 7.5 (+5.8) | 7.7 (+5.7) |
| Triglycerides | 0.7 (+0.4) | 0.7 (+0.5) | 0.9 (+0.5) | 0.9 (+0.5) |
| HDL-C | 1.4 (+0.3) | 1.4 (+0.4) | 1.3 (+0.3) | 1.3 (+0.4) |
| Systolic BP | 115 (+12) | 115 (+12) | 114 (+10) | 114 (+10) |
| Diastolic BP | 68 (+10) | 68 (+9.5) | 59 (+6) | 59 (+6) |
| Anxious-depressed | 15 (+4) | 15 (+4) | 17 (+4) | 16 (+5) |
| Withdrawn depressed | 9 (+3) | 9 (+3) | 10 (+2) | 10 (+4) |
| Somatic complaints | 13 (+4) | 13 (+4) | 13 (+3) | 13 (+5) |
| ***Parental measures during pregnancy*** |  |  |  |  |
| Maternal pre-pregnancy BMI | 21.7 (+3.7) | 21.7(3.5) | 22.2 (+3.8) | 22.5 (+4.5) |
| Maternal end-pregnancy BMI | 26.9 (+4.4) | 27.1 (+4) | 27.3 (+4.9) | 27.6 (+4.6) |
| Maternal age | 27 (+7) | 28 (+7) | 29 (+5.8) | 28.9 (+5.8) |
| Marital status (Unmarried/single) | 251 (+3.7%) | 15 (+2.8%) | 164 (13.3%) | 120 (12.1%) |
| Nulliparous | 2165 (+32.7%) | 173 (+32.8%) | 485 (51.3%) | 367 (48%) |
| Maternal smoking | 416 (+6.9%) | 31 (+6.4%) | 246 (19.9%) | 232 (23.4%) |
| Maternal alcohol use | 752 (+11.6%) | 54 (+10.3%) | 607 (49.2%) | 489 (49.2%) |
| Paternal smoking | 2212 (+36.7%) | 169 (+34.5%) | 398 (32.7%) | 354 (35.7%) |

Note: Values are percentages for categorical variables, mean (+SD) for continuous variables with normal distribution and median (IR) for skewed variables. Abbreviations: BMI: Body Mass Index, HDL-C: High Density Lipid - Cholesterol


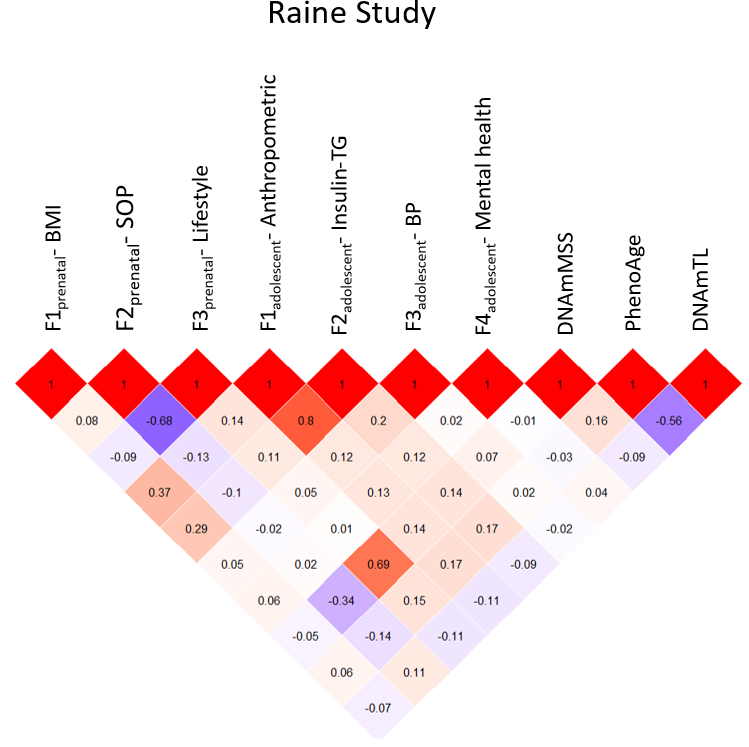


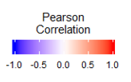

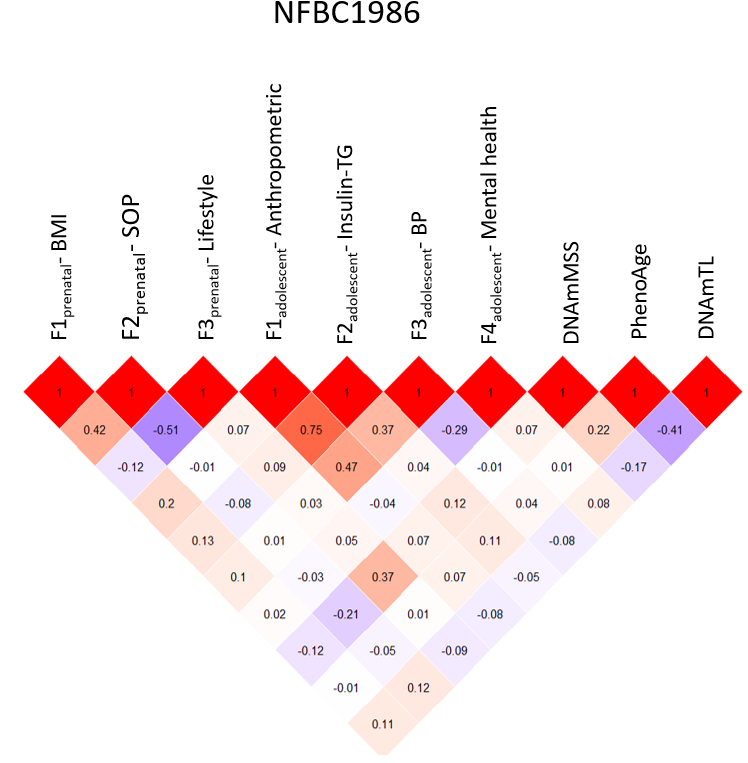


# **Fig S1.** Correlation between prenatal and adolescent psycho-cardiometabolic latent factors with epigenetic biomarkers: DNAmMSS, PhenoAge and DNAmTL.

Abbreviations: BMI: Body Mass Index; BP: Blood Pressure; DNAmMSS: DNA methylation age smoking score; DNAmTL: DNA methylation age telomere length; NFBC1986: Northern Finland Birth Cohort 1986; SOP: Socio-obstetric profile; TG: Triglycerides.
